# The prevalence of depression and associated risk factors among medical students: An untold story in Vietnam

**DOI:** 10.1371/journal.pone.0221432

**Published:** 2019-08-20

**Authors:** Tung Pham, Linh Bui, Anh Nguyen, Binh Nguyen, Phung Tran, Phuong Vu, Linh Dang

**Affiliations:** 1 Center for Population Health and Sciences, Hanoi University of Public Health, Hanoi, Vietnam; 2 Friendship and Science for Health Research group, Dinh Tien Hoang Institute of Medicine, Hanoi, Vietnam; 3 Department of Physiology, Hanoi Medical University, Hanoi, Vietnam; 4 Department of Obstetrics and Gynecology, Hanoi Medical University, Hanoi, Vietnam; 5 Institute of Gastroenterology and Hepatology, Hanoi, Vietnam; 6 Laboratory Center, Hanoi University of Public Health, Hanoi, Vietnam; Uniformed Services University of the Health Sciences, UNITED STATES

## Abstract

**Background:**

Depression is a common mental health problem in medical students worldwide. The association between depression and motivation in Vietnamese medical students is not well-documented.

**Objectives:**

To estimate the prevalence of self-reported depression and to identify associated risk factors among medical students at Hanoi Medical University (HMU).

**Method:**

A cross-sectional study was conducted on medical students with clinical experience at HMU from November 2015 to January 2016. We used the multistage cluster random sampling technique to select and invite students to complete a questionnaire including demographic characteristics, Patient Health Questionnaire 9 (PHQ-9), Academic Motivation Scale (AMS), and International Physical Activity Questionnaire Short Form (IPAQ).

**Results:**

Among 494 participants (78.8% response rate), the prevalence of self-reported depression was 15.2% (95%CI:12.0%-19.0%), and suicidal ideation was 7.7% (95%CI:6.2%-9.5%). Self-reported depression was significantly associated with perceived financial burden, physical inactivity, being senior student, perceived negative influence of night shifts, and non-self-determined motivation profile. Suicidal ideation was significantly associated with perceived financial burden and non-self-determined motivation profile. In the multivariable regression models, significant risk factors for self-reported depression were non-self-determined motivation (PR = 2.62, 95%CI:1.68–4.07), perceived financial burden (PR = 1.95, 95%CI:1.39–2.73), and vigorous level of physical activity (PR = 0.43, 95%CI:0.20–0.942). For suicidal ideation, non-self-determined motivation (PR = 2.33, 95%CI:1.13–4.80) and perceived financial burden (PR = 1.91, 95%CI:1.16–3.13) were significant risk factors.

**Strengths and limitations:**

The strengths of our study included a representative sample, a good response rate, and using a good depression screening tool. However, the PHQ-9 only allowed us to screen for depression, and the translation of the AMS and IPAQ into Vietnamese could potentially decrease these tools’ validity.

**Conclusion:**

The prevalence of self-reported depression and suicidal ideation in medical students is notably higher compared to the general population in Vietnam. Non-self-determined motivation and financial burden were the prominent risk factors for both the depression and suicidal ideation in medical students.

## Introduction

Depression is one of the most common mental health issues among medical students worldwide due to the high intensity of training[1]. Recent systematic reviews and meta-analysis indicated that the prevalence of depression among medical students worldwide was around 28%, and suicidal ideation was 11.1%[2–4]. In Vietnam, Quynh Anh et al. also surveyed medical students at 8 different Vietnam medical universities using the Center for Epidemiologic Studies Depression Scale (CES-D) questionnaire in 2014[5,6]. The result showed that the prevalence of depressive symptoms was 43.2%, and suicidal ideation was 8.7%[5,6]. This prevalence of depression was much higher compared to the general Vietnamese population, of which the proportion with depression symptoms is only 2.8% according to the Ministry of Health[7].

The burden of depression on medical students could lead to low quality of life, dropout[8], and ultimately suicidal ideation; therefore, identifying risk factors for depression among this group should be a priority. Quynh Anh et al. identified several potential risk factors for depression including financial burden, stress associated with exams, prolonged study period at medical schools, and so forth[5,6]. However, there were other factors remained undocumented in the literature, such as the motivation of students toward studying medicine. The clinical application of the CES-D questionnaire was also limited with a positive Likelihood Ratio (LR), which is a good measure of diagnostic accuracy, ranging from 3 to 4 in the general population depending on the cut-off[9]. Smaller studies in Vietnamese medical students employed other questionnaires such as the Beck Depression Inventory—Short Form[10] and Depression, Anxiety and Stress Scales (DASS-21)[11]. These questionnaires also had similar positive LR, ranging from 3 to 5, compared to the CES-D in the general population and medical settings[12–14]. Therefore, a new study which utilizes a better depression screening method with higher positive LR is genuinely needed.

While medical schools in other developed countries are employing a holistic admission process[15–19] that takes test scores, motivational essays, and structured interviews into account, many medical universities in China, India, and other low-middle income countries, such as Vietnam, Thailand, Bangladesh, Nepal, Ghana, and Kenya, still recruit their students based solely on the university entrance exam score[20–28]. Students’ motivation, though playing an important role in their academic performance and well-being, has never been formally and structurally evaluated in Vietnam. As a result, many students simply enroll at a university due to external factors, such family influences and social norms[29,30]. Therefore, it is necessary to re-assess the depression using a more reliable tool as well as explore other potential related risk factors among medical students in Vietnam.

In this study, we aimed to estimate the prevalence of self-reported depression and suicidal ideation among medical students at Hanoi Medical University in Vietnam and to examine the association between various risk factors, such as motivation, and depression in medical students at Hanoi Medical University.

## Method

### Study design and setting

We conducted a cross-sectional study on medical students from the general practitioner track, who were exposed to the clinical environment (4^th^, 5^th^, and 6^th^ year), at Hanoi Medical University (HMU) from November 2015 to January 2016. Medical graduates from the general practitioner track are trained to provide primary care in Vietnam or continue specialist training after graduation; the detailed description of medical education system in Vietnam was presented elsewhere[23]. HMU is one of the largest, public medical schools in Vietnam with a 6-year curriculum for medical graduates. There are around 550 medical students in each year of the program. The training is divided into two parts: (1) basic science in the first three years; and (2) clinical science and rotations in the last three years[23].

Our research proposal and protocol were approved by the Institutional Review Board of Hanoi University of Public Health with the registration number: 280/2015/YTCC-HD3. All invited students were asked for written informed consent after reading the study description and understanding the minimal risks involved with the participation as well as the confidentiality of their data. All participation was voluntary, and the subjects could leave the study at any time. When a student agreed to participate in our survey, he or she could choose to participate in a lottery to win an incentive, which was one of the three Littmann stethoscopes. Contact information for the lottery was provided separately from the study questionnaire.

### Sample and data collection

We used the multistage cluster sampling technique to select our participants from medical students in three cohorts (4^th^, 5^th^, and 6^th^ year). First, we randomly selected 4 out of 6 classes within each cohort; then, in each class, 2 out of 4 groups would be randomly selected. Finally, we had a sampling frame of 627 students. The invitations were sent via group emails and/or face-to-face interaction with the students at the school, hospitals, dormitories, and so forth. Invited students, who agreed to participate, were then asked to complete a self-reported paper-based questionnaire. We did not collect any personal identifiable information to encourage the willingness to participate and avoid social desirability bias among the students. After collecting the questionnaires, the investigators entered and encrypted all data into a password-protected computer. Because it was an anonymous survey, we could not create a list of students with potential mental health problems for further follow-up with psychological support services. Furthermore, at the time of our study, there was no general student support/ counseling services or mental health services for both students and staff at HMU as well as many other universities in Vietnam.

### Survey instruments

The survey instruments included questions on demographic information, socioeconomic status, lifestyle (alcohol consumption, smoking status, International Physical Activity Questionnaire Short Form (IPAQ-SF)), academic performance (grade point average—GPA), number of night shifts, history of failing a theoretical/clinical test), the Patient Health Questionnaire (PHQ-9) to screen for depressive symptoms, and the Academic Motivation Scale (AMS).

GPA was reported according to the Vietnamese grading system on a scale from 1 to 10 points; the conversion and explanation of the grading system were described elsewhere[31]. In brief, the score range can be explained as follow: below 5 –failed, the student needs to retake the exam; from 5 to 6 –average; from 6 to 7– fairly good; from 7 to 8 –good; from 8 to 9 –good; and from 9 to 10 –excellent. A pilot study on 52 medical students, who were selected by convenience sampling, was also conducted to test the readability and appropriateness of our survey.

The usage and validity of PHQ-9 have been well described in other countries[32,33] and in Vietnam[34,35]. A validated Vietnamese version of PHQ-9 was used to screen for depressive symptoms in our study population. The PHQ-9 was aligned with the diagnostic criteria of DSM-V and consisted of 9 questions with the range of score from 0 to 27[36]. Each question asked how frequently the subject had been bothered by any of mental-related symptoms in the last two weeks. The subject would choose 0- “Not at all”, 1- “Several days”, 2- “More than half the days”, or 3- “Nearly every days” for each question. We applied the recommended cut-offs to categorize depression severity: none/minimal (0–4), mild (5–9), moderate (10–14), moderately severe (15–19), and severe (20–27)[36]. The sensitivity and specificity were reported as well-balanced at the cut-off point of 10[32,33,37], and a meta-analysis showed a sensitivity of 80% and specificity of 92% with this cut-off[38]. The positive LR of the PHQ-9 was also higher than 10 when using this approach[38]. Therefore, we categorized participants scoring 10 points and above as screened positive for depressive symptoms.

Reporting suicidal ideation was defined as receiving a score from 1 and above on item 9 of the questionnaire, which was “Over the last two weeks, how often have you had thoughts that you would be better off dead or hurting yourself?”[37]. Although the utilities and validity of the item 9 in the PHQ-9 was lower compared to other well-established instruments, such as the Columbia-Suicide Severity Rating Scale[39], the Joint Commission recommended that item 9 could be used to screen for suicidal ideation and suicide risks in medical settings[40]. Many studies also reported that a score of 1 and above on item 9 of the PHQ-9 was also associated with an increased risk for suicide attempt or death[41–43].

The Academic Motivation Scale (AMS) was designed by Vallerand et al. to measure the motivation to education[44] and was used to calculate the self-determination index (SDI) for each student[45–49]. The conceptual framework of AMS was based on Deci and Ryan’s Self-Determination Theory[50]. The questionnaire contained 28 statements assessing intrinsic motivation (to know, to accomplish things, and to experience stimulation), extrinsic motivation (external, introjected, and identified regulation), and a lack of motivation—amotivation[44]. Students would report their agreement on each statement using a 7-point Likert scale (1- “Does not correspond at all”; 2 and 3- “Corresponds a little”; 4- “Corresponds moderately”; 5 and 6- “Corresponds a lot”; 7- “Corresponds exactly”)[44]. SDI score of each question groups would be weighted using the formula: [(2×Intrinsic Motivation) + (1×Identified Regulation)–(0.5×External Regulation)–(1×Introjected Regulation)–(2×Amotivation)][45–49]. As a result, the total SDI score could range from -18 (little self-determined) to +18 (extreme self-determined) and would reflect where the students’ motivation was on the self-determination continuum–from amotivation to intrinsic motivation[45–49,51]. Therefore, SDI was frequently used to measure and compared motivation among various groups[45–49]. Based on SDI, the students were divided into two groups: self-determined academic motivation profile (SDI > 0 –closer to intrinsic motivation) and non-self-determined academic motivation profile (SDI ≤ 0 –closer to amotivation)[45–49].

The International Physical Activity Questionnaire (IPAQ) has been widely used in many countries[52,53]. The authors of IPAQ recommended using the 9-item short form (IPAQ-SF) for monitoring purposes and to reduce the burden on participants[52,54]. Therefore, we decided to use the self-administered adult version of the IPAQ-SF, which was designed to collect information from individuals aged 15–69 years. The short-form questionnaire contained 9 questions on vigorous, moderate activities, walking, and sitting time in the last 7 days. The contents, usage, and scoring of the IPAQ-SF have been described elsewhere[52,54].

Finally, a physician with WHO-certified English-Vietnamese translation skills translated all English questionnaires including AMS and IPAQ-SF, of which validated Vietnamese versions were not available, into Vietnamese. Another independent physician back translated those Vietnamese documents into English, and then, another third professional translator compared two English versions to make sure they were comparable in terms of meaning. All the questionnaires through this process were modified modestly to adapt to Vietnamese culture and language.

### Data analysis

Stata 13.1 Survey package was used to adjust for cluster sampling and calculate the prevalence of depression and suicidal ideation[55,56]. The Chi-squared, Fisher exact, Wilcoxson, and Kruskal–Wallis test were then used to compare the difference among three cohorts of students, depression severities, and suicidal ideation/non-suicidal ideation students.

With the high prevalence of depression among our study population, logistic regression would overestimate the association of independent variables with this binary outcome[57,58]. Using log-binomial regression models to direct estimate Prevalence Ratios (PRs) would be preferable in this case; however, this type of models often fails to converge[59]. Zou et al. and Barros et al. reported that a modified Poisson regression model (Poisson regression with a robust error variance) with binary outcome data could be used to calculate PRs (or relative risk with cohort study) [57,58]. Chen et al. also confirmed that log-binomial and modified Poisson regression models produced similar results[60]. Therefore, we calculated PRs using the modified Poisson regression model with cluster sampling adjustment using the Stata survey—svy—commands to assess the association of potential risk factors with our outcome of interest[55,57,58].

R statistical software version 3.4.0 and forest plot package were used to create the forest plots after the models were produced with Stata 13.1[56,61,62].

## Results

Among 627 invited candidates, 494 (78.8%) students, who agreed to participate and completed the questionnaires, were included in the analysis. Among those included, 32 participants did not complete the IPAQ-SF portion of the survey.

**Table 1** shows that there were no significant characteristic differences among the three cohorts. The proportion of males was consistently slightly higher than that of females in all cohorts. About 70% of the students lived with roommate(s) at the time of our survey. Regarding lifestyle factors, one in five students reported drinking alcohol more than once a month (or more than twelve times a year). 98% of students had never smoked, and the year-six cohort had more smokers than other cohorts (p = 0.051). We also found that over 80% of students reported a moderate-to-vigorous activity level within the last 7 days, and there was no difference regarding physical activity among cohorts. Nearly one fifth of our participants were characterized as non-self-determined motivated by the AMS questionnaire, and there was no difference among cohorts (p = 0.77).

**Table 1:** Characteristics of participants.

Variables	Total	4th year	5th year	6th year	p-value
	n = 494	n = 160	n = 166	n = 168	
**Demographic**					
**Gender**					0.39^a^
Male	277 (56.1%)	88 (55.0%)	100 (60.2%)	89 (53.0%)	
Female	217 (43.9%)	72 (45.0%)	66 (39.8%)	79 (47.0%)	
**Age**					**<0.001**^**b**^
21	149 (30.2%)	148 (92.5%)	1 (0.6%)	0 (0.0%)	
22	169 (34.2%)	10 (6.3%)	158 (95.2%)	1 (0.6%)	
23	163 (33.0%)	1 (0.6%)	6 (3.6%)	156 (92.9%)	
24 and above	13 (2.6%)	1 (0.6%)	1 (0.6%)	11 (6.5%)	
**Types of housemate**					0.056^a^
Living with family	94 (19.0%)	38 (23.8%)	20 (12.0%)	36 (21.4%)	
Living alone	56 (11.3%)	14 (8.8%)	21 (12.7%)	21 (12.5%)	
Living with roommate(s)	344 (69.6%)	108 (67.5%)	125 (75.3%)	111 (66.1%)	
**Lifestyle factors**					
**Alcohol drinking**					0.75^a^
Less than once a month	386 (78.1%)	127 (79.4%)	131 (78.9%)	128 (76.2%)	
More than once a month	108 (21.9%)	33 (20.6%)	35 (21.1%)	40 (23.8%)	
**Smoking status**					0.051^b^
Never	482 (97.6%)	159 (99.4%)	163 (98.2%)	160 (95.2%)	
Ever	12 (2.4%)	1 (0.6%)	3 (1.8%)	8 (4.8%)	
**Physical activity level**					0.17^a^
Low	78 (15.8%)	34 (21.3%)	22 (13.3%)	22 (13.1%)	
Moderate	254 (51.4%)	74 (46.3%)	88 (53.0%)	92 (54.8%)	
Vigorous	130 (26.3%)	45 (28.1%)	46 (27.7%)	39 (23.2%)	
Unknown	32 (6.5%)	7 (4.4%)	10 (6.0%)	15 (8.9%)	
**Academic factors**					
**GPA of the previous year**	7.67 (7.35, 7.99)	7.69 (7.36, 7.99)	7.65 (7.25, 7.96)	7.67 (7.38, 8.00)	0.70^c^
**Academic motivation profile**					0.77^a^
Self-determined	397 (80.4%)	130 (81.3%)	135 (81.3%)	132 (78.6%)	
Non-self-determined	97 (19.6%)	30 (18.8%)	31 (18.7%)	36 (21.4%)	

Grade point average (GPA—ranging from 0 to 10) is presented as median (interquartile range); other variables are presented as number (percentage).

Statistical comparison using ^a^ Chi-square test, ^b^ Fisher's exact test, and ^c^ Kruskal–Wallis test. Unknown category was not used for comparison for physical activity levels.

The bold p-value indicated statistical significance (p<0.05).

The proportion of students screened positive for depressive symptoms (from moderate to severe categories) was 15.2% (95% CI: 12.0%-19.0%) and suicidal ideation was 7.7% (95% CI: 6.2%-9.5%) **(Table 2)**. The distribution of depression severity was as follows: Moderate: 12.0% (95% CI: 9.4%-15.0%); Moderately Severe: 2.6% (95% CI: 1.8%-3.9%); and Severe: 0.6% (95% CI: 0.2%-1.6%). Students who reported depression were more likely to perceive financial burden (45.3% vs. 27.2%, p < 0.001) and less likely to be physically active compared to the other group (p = 0.032). **In Table 2**, We also observed a significant difference in depression prevalence among three cohorts of students, with Year 6 having the highest number of students screened positive for depressive symptoms (Year 6: 45.3% vs. Year 5: 22.7% vs. Year 4: 32.0%, p = 0.039). There were also significant differences among depression severity profiles regarding the number of students perceiving negative effects of night shifts (p < 0.001) and identified as non-self-determined using the AMS scale (p < 0.001). The proportion of students reporting suicidal ideation (using item 9 of the PHQ-9 - “Over the last two weeks, how often have you had thoughts that you would be better off dead or hurting yourself?”) was 13.7 times higher in the group reported depression compared to the normal group (36.0% vs. 2.6%). Consistent with previous findings, students reporting suicidal ideation were also more likely to perceive financial burden (p = 0.038) and to have a non-self-determined motivation profile (p = 0.001).

**Table 2:** Prevalence of depression and suicidal ideation.

Variables	Depression severity			p-value	Suicidal ideation	p-value
	None/Minimal	Mild	Moderate to Severe			
	n = 259	n = 160	n = 75		n = 38	
**Prevalence (95%CI)**	52.4% (48.6%-56.3%)	32.4% (28.9%-36.1%)	15.2% (12.0%-19.0%)		7.7% (6.2%-9.5%)	
**Demographic**						
**Gender**				0.68^a^		0.21^a^
Male	150 (57.9%)	87 (54.4%)	40 (53.3%)		25 (65.8%)	
Female	109 (42.1%)	73 (45.6%)	35 (46.7%)		13 (34.2%)	
**Types of housemate**				0.61^a^		0.96^b^
Living with family	46 (17.8%)	35 (21.9%)	13 (17.3%)		8 (21.1%)	
Living alone	26 (10.0%)	19 (11.9%)	11 (14.7%)		4 (10.5%)	
Living with roommate(s)	187 (72.2%)	106 (66.3%)	51 (68.0%)		26 (68.4%)	
**Perceived financial burden**				**<0.001**^**a**^		**0.038**^**a**^
No	203 (78.4%)	102 (63.7%)	41 (54.7%)		21 (55.3%)	
Yes	56 (21.6%)	58 (36.3%)	34 (45.3%)		17 (44.7%)	
**Lifestyle factors**						
**Alcohol drinking**				0.17^a^		0.27^a^
Less than once a month	208 (80.3%)	117 (73.1%)	61 (81.3%)		27 (71.1%)	
More than once a month	51 (19.7%)	43 (26.9%)	14 (18.7%)		11 (28.9%)	
**Smoking status**				0.069^b^		0.23^b^
Never	256 (98.8%)	155 (96.9%)	71 (94.7%)		36 (94.7%)	
Ever	3 (1.2%)	5 (3.1%)	4 (5.3%)		2 (5.3%)	
**Physical activity level**				**0.032**^**a**^		0.94^a^
Low	36 (13.9%)	28 (17.5%)	14 (18.7%)		5 (15.6%)	
Moderate	133 (51.4%)	75 (46.9%)	46 (61.3%)		17 (53.1%)	
Vigorous	78 (30.1%)	43 (26.9%)	9 (12.0%)		10 (31.3%)	
Unknown	12 (4.6%)	14 (8.8%)	6 (8.0%)			
**Academic factors**						
**Year in medical school**				**0.039**^**a**^		0.17^a^
4th year	80 (30.9%)	56 (35.0%)	24 (32.0%)		11 (28.9%)	
5th year	101 (39.0%)	48 (30.0%)	17 (22.7%)		9 (23.7%)	
6th year	78 (30.1%)	56 (35.0%)	34 (45.3%)		18 (47.4%)	
**GPA of the previous year, median (IQR)**	7.68 (7.38, 8.01)	7.645 (7.3, 8.00)	7.66 (7.35, 7.92)	0.78^d^	7.69 (7.35, 7.91)	0.87^c^
**Failing a test in medical school**				0.19^a^		0.076^a^
Never	127 (49.0%)	68 (42.5%)	29 (38.7%)		12 (31.6%)	
Ever	132 (51.0%)	92 (57.5%)	46 (61.3%)		26 (68.4%)	
**Negative effects of night shifts on academic performance and quality of life**				**<0.001**^**a**^		0.16^a^
No	158 (61.0%)	72 (45.0%)	32 (42.7%)		16 (42.1%)	
Yes	101 (39.0%)	88 (55.0%)	43 (57.3%)		22 (57.9%)	
**Academic motivation profile**				**<0.001**^**a**^		**0.001**^**a**^
Self-determined	225 (86.9%)	128 (80.0%)	44 (58.7%)		23 (60.5%)	
Non-self-determined	34 (13.1%)	32 (20.0%)	31 (41.3%)		15 (39.5%)	
**Suicidal ideation**	** **	** **	** **	**<0.001**^**b**^	** **	** **
No	258 (99.6%)	150 (93.8%)	48 (64.0%)			
Yes	1 (0.4%)	10 (6.3%)	27 (36.0%)			

Grade point average (GPA—ranging from 0 to 10) is presented as median (interquartile range); other variables are presented as number (percentage).

Statistical comparison using ^a^ Chi-square test, ^b^ Fisher's exact test, ^c^ Wilcoxon rank-sum test, and ^d^ Kruskal–Wallis test. Unknown category was not used for comparison for physical activity levels.

Bold p-values indicated statistical significance (p<0.05).

**Fig 1:**
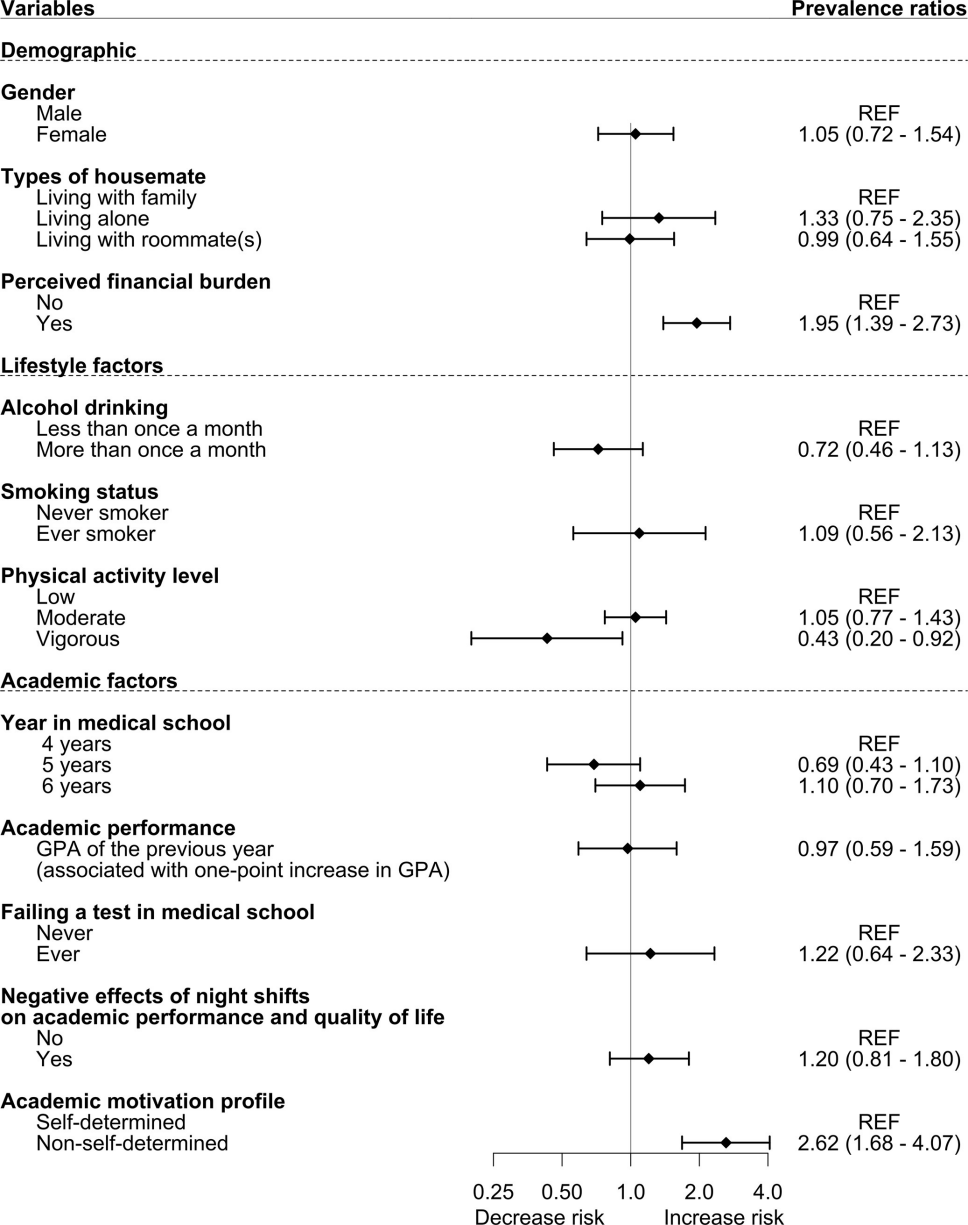
Risk factors for depression. All PRs were adjusted using the Poisson multivariable regression model.

**Fig 2:**
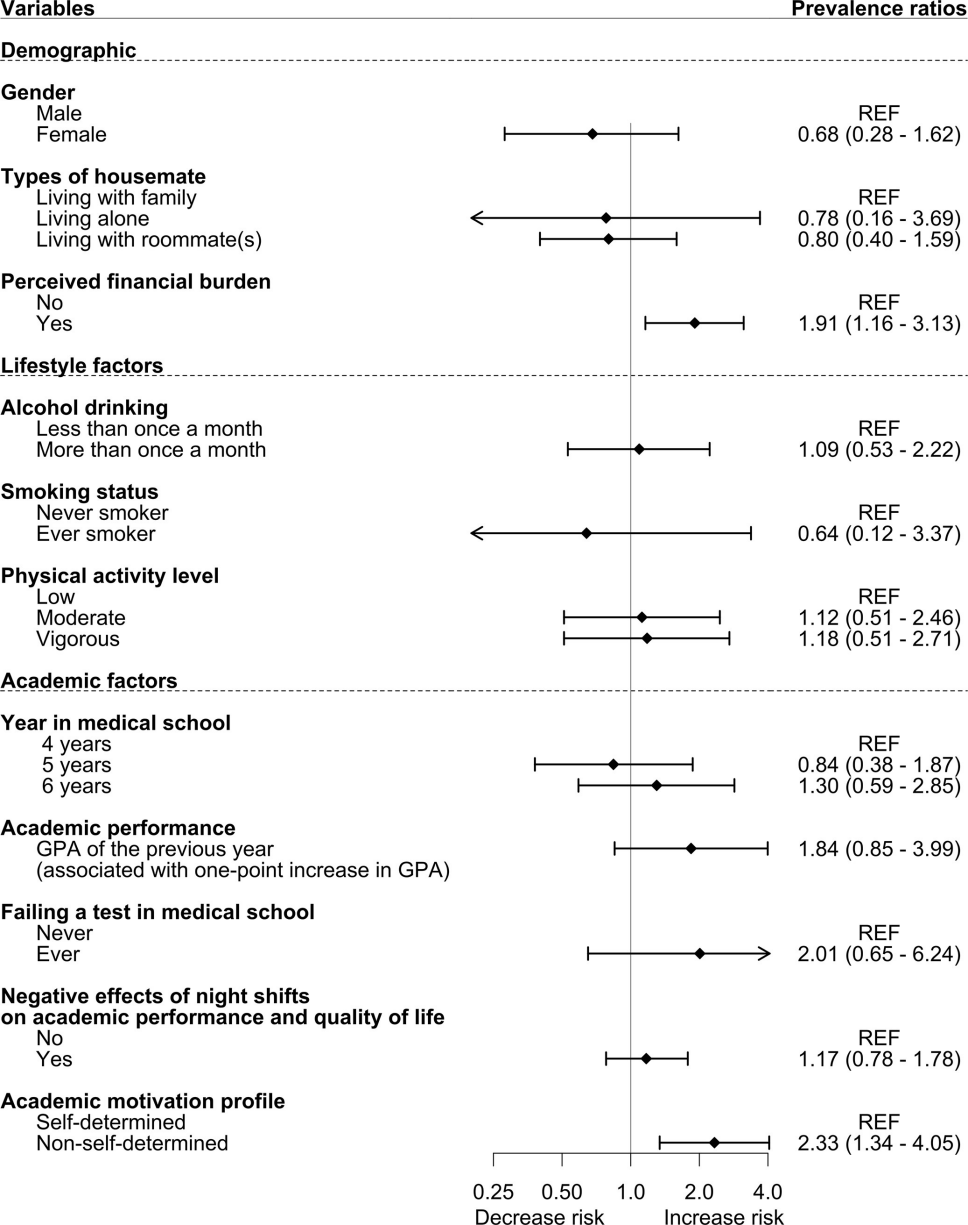
Risk factors for suicidal ideation. All PRs were adjusted using the Poisson multivariable regression model.

In the Poisson multivariable regression model **(Fig 1)**, independent variables that showed significant association with self-reported depression included non-self-determined motivation profile (PR = 2.62, 95% CI: 1.68–4.07), perceived financial burden (PR = 1.95, 95% CI: 1.39–2.73), and vigorous physical activity level (PR = 0.43, 95% CI: 0.20–0.92). Regarding self-reported suicidal ideation **(Fig 2)**, non-self-determined motivation profile (PR = 2.33, 95% CI: 1.13–4.80) and perceived financial burden (PR = 1.91, 95% CI: 1.16–3.13) were the only variables that demonstrated significant association. Additional sensitivity analyses were conducted to check if the missingness of physical activity variables (32 participants) would affect the associations and did not find any meaningful differences in the regression models.

## Discussion

Our study showed that among 494 surveyed medical students, 15.2% were screened positive for depressive symptoms and 7.7% reported suicidal ideation. The prevalence of self-reported depression among our sample is more than five times higher compared to the general population in Vietnam (15.2% vs. 2.8%)[7]. Regarding suicidal ideation, Huong et al. in 2006 using the WHO SUPRE-MISS community survey questionnaire[63] reported that the prevalence of lifetime suicidal ideation, suicide plans, and suicidal attempt in the general Vietnamese population were 8.9%, 1.1%, and 0.4%, respectively[64]. Our prevalence of suicidal ideation (7.7%) was lower than the prevalence of lifetime suicidal thought in general population probably because the PHQ-9 focused on self-reported suicidal ideation only within the last two weeks[36,37].

The prevalence of depression in our study was nearly 30% lower than the prevalence from Quynh Anh et al. study on Vietnamese medical students in 2014[5,6]. This discrepancy could be partly explained by the sampling selection and specific assessment tools used to screen for depression as one systematic review has pointed out[65]. Quynh Anh et al. focused on the prevalence of depression at different medical schools, so the study could only collect data of around 240 students (80 for each of 1^st^, 3^rd^, and 5^th^ year) from each school using the CES-D questionnaire[5,6]. Our study, on the other hand, concentrated the efforts on HMU students who had clinical experiences (4^th^, 5^th^, and 6^th^ year) using the PHQ-9 questionnaire. While the sensitivities of both PHQ-9 and CES-D were high for depression screening (80 vs. 83%) [9,38], the specificity of PHQ-9 was noticeably higher than CES-D (92% vs. 78%)[9,38]. Therefore, the positive LR of PHQ-9 was greater than 10[9,38], which was a strong evidence to rule in depression[66,67], compared to CES-D’s positive LR of just 3 to 4[9,38]. Moreover, our instrument—the PHQ-9 was closely aligned with DSM-V depression diagnostic criteria that are widely used and recommended in clinical practice[32,36]. In comparison with other countries, the prevalence of students reported depression in our study (15.2%) was comparable to studies in Mexico (16.2%), United States (14.3%), New Zealand (16.9%) and South Korea (13.8%), which also used PHQ-9 ≥ 10 to identify students with depression [3,68–71].

We also found that 19.6% of surveyed medical students at HMU were identified as individuals with non-self-determined motivation. This finding was supported by previous studies that family influences and social norms play important roles in Vietnamese students’ decision to enroll at a university[29,30]. Meanwhile, Quynh Anh et al. also reported that 57% of Vietnamese medical students were not satisfied with their academic achievement, and 27.7% of them reported having second thoughts about a medical career[6]. Such students could be more likely to drop-out or not pursuing clinical practice after graduation compared to self-determined students[8,72,73]. This finding should raise a considerable concern over the improvement of the admission process as well as the development of student mental, academic, and career support services. Due to the shortage of medical professionals in Vietnam, all medical schools are encouraged to accept a higher number of students to meet the increasing demand of the healthcare system[74]. In 2015, with the current proportion of 6.7 physicians per 10.000 citizens in Vietnam, the Partnership for Health Advancement in Vietnam (HAIVN) recommended that all graduated medical students have to practice medicine for a certain period of time to create enough physicians for Vietnam in the future[75]. However, expanding the medical program would not solve the cause of healthcare worker shortage if many of these students do not have the motivation to pursue medical training and passion for a medical career after graduation.

In our regression models, we found that academic motivation was the most important factors associated with self-reported depression (PR = 2.62, 95% CI: 1.68–4.07) and suicidal ideation (PR = 2.33, 95% CI: 1.34–4.05). Quynh Anh et al. also pointed out that Vietnamese medical students, who considered to re-select their career paths and pursued medical career according to his/her family’s wish, had higher odds of reporting depression and suicidal ideation compared to students who did not have that consideration[5,6]. These results were consistent with previous findings that students who are genuinely motivated with their career choice would be more able to cope with the intense medical training[76,77] and ultimately avoid being depressed compared to non-motivated students. Other cross-sectional studies in students worldwide also reported the positive association of motivation and quality of life[78], academic achievements[78], and inverse association of motivation and depression[79]. On the other hand, a cohort study indicated that positive motivation to important life goals was a protective factor against depression among young adults[80]. These consistent findings strengthen our hypothesis that the association of motivation and depression was prominent and may suggest a causal relationship that needs to be examined in future studies.

Consistent with previous studies, perceived financial burden was found to be a major factor associated with self-reported depression (PR = 1.95, 95% CI: 1.29–2.94 in our study) and suicidal ideation (PR = 1.91, 95% CI: 1.16–3.13 in our study)[5,81]. At the time this study was conducted, there was no official financial assistance and scholarship program from the Vietnamese government that could keep up with the rising tuition fees[82,83] and living costs, especially in urban areas. This situation has created a tremendous burden on disadvantaged students. As more and more medical schools were required to be financially independent from the government[84], we could expect tuition fees to increase rapidly in the near future. Therefore, an appropriate financial aid package is needed not only to decrease the depression burden but also to ensure equity in accessing medical education in Vietnam.

We also found that vigorous physical activity showed an inverse association with self-reported depression (PR = 0.43, 95% CI: 0.20–0.92 in our study), which was also consistent with previous findings[85–88]. Moreover, recent reviews pointed out that exercise has positive effects on patients with depression in many intervention studies [85,87,88]. The American Psychiatric Association (APA) also stated that physical activity could be a reasonable addition to depressive disorder treatment plan[89]. These findings suggest that future studies may need to focus on promoting physical activity at an appropriate level in medical school and assess its effect on the mental health of students.

The strength of our study was that the cluster random sampling technique could enhance the representativeness of our sample when calculating the self-reported depression and suicidal ideation prevalence among the study population. We achieved a good response rate, which improved the internal validity of our study. Moreover, the PHQ-9 questionnaire used in our survey was closely aligned with clinical diagnostic criteria with high sensitivity and specificity. HMU was also similar with other medical schools in Vietnam in terms of admission process and training curriculum at the time of this study, so we believe that our results could be generalizable to other medical universities in Vietnam. However, the PHQ-9 questionnaire only allows us to screen for depressive symptoms but could not confirm the clinical diagnosis, and the item 9 of the PHQ-9 is also an inferior tool when used to screen for suicidal ideation compared to other specific instruments. The anonymous nature of the survey did not allow us to follow up the students who presented with depressive symptoms and suicidal ideation, and we were not able to collect the information on prior history of depression or other mental health conditions before entering the medical school. The Vietnamese translation and minimal modification of other English questionnaires could potentially decrease the validity of these instruments in our study. Moreover, the cross-sectional design also limited our interpretation of the results to association rather than causation. We believe that a prospective cohort and perhaps an intervention study would be needed to further explain our findings and provide more evidence to the policy makers regarding medical schools’ admission and mental health support for students in Vietnam.

In conclusion, the burden of depression and suicidal ideation among medical students in Vietnam is much higher than the general population of Vietnam. The most prominent risk factors for both self-reported depression and suicidal ideation were non-self-determined motivation and financial burden. More studies are needed to explore the essential role of motivation and financial burden in mental health of medical students in Vietnam to provide appropriate consulting and supporting programs.

## Supporting information

S1 Table Depression severity among medical students. (DOCX)

S1 File Questionnaire. (PDF)
