# Supplementary material for: The prevalence of depression and associated risk factors among medical students: An untold story in Vietnam
Source: PLoS One. 2019 Aug 20;14(8):e0221432. doi: 10.1371/journal.pone.0221432 (PMC6701769; doi:10.1371/journal.pone.0221432)
Supplement: S1 Table — (DOCX) [file pone.0221432.s001.docx]

**S1 Table.** **Depression severity among medical students**

| **Variables** | **Depression severity** | | | | |
| --- | --- | --- | --- | --- | --- |
|  | **None/Minimal** | **Mild** | **Moderate** | **Moderately severe** | **Severe** |
|  | **n = 259** | **n = 160** | **n = 59** | **n = 13** | **n = 3** |
| **Prevalence**  **(95%CI)** | 52.4%  (48.6%-56.3%) | 32.4%  (28.9%-36.1%) | 11.9%  (9.4%-15.0%) | 2.6%  (1.8%-3.9%) | 0.6%  (0.2%-1.6%) |
| **Demographic** |  |  |  |  |  |
| **Gender** |  |  |  |  |  |
| Male | 150 (57.9%) | 87 (54.4%) | 32 (54.2%) | 6 (46.2%) | 2 (66.7%) |
| Female | 109 (42.1%) | 73 (45.6%) | 27 (45.8%) | 7 (53.8%) | 1 (33.3%) |
| **Types of housemate** |  |  |  |  |  |
| Living with family | 46 (17.8%) | 35 (21.9%) | 7 (11.9%) | 5 (38.5%) | 1 (33.3%) |
| Living alone | 26 (10.0%) | 19 (11.9%) | 8 (13.6%) | 3 (23.1%) | 0 (0.0%) |
| Living with roommate(s) | 187 (72.2%) | 106 (66.3%) | 44 (74.6%) | 5 (38.5%) | 2 (66.7%) |
| **Perceived financial burden** |  |  |  |  |  |
| No | 203 (78.4%) | 102 (63.7%) | 31 (52.5%) | 8 (61.5%) | 2 (66.7%) |
| Yes | 56 (21.6%) | 58 (36.3%) | 28 (47.5%) | 5 (38.5%) | 1 (33.3%) |
| **Lifestyle factors** |  |  |  |  |  |
| **Alcohol drinking** |  |  |  |  |  |
| Less than once a month | 208 (80.3%) | 117 (73.1%) | 50 (84.7%) | 9 (69.2%) | 2 (66.7%) |
| More than once a month | 51 (19.7%) | 43 (26.9%) | 9 (15.3%) | 4 (30.8%) | 1 (33.3%) |
| **Smoking status** |  |  |  |  |  |
| Never | 256 (98.8%) | 155 (96.9%) | 57 (96.6%) | 12 (92.3%) | 2 (66.7%) |
| Ever | 3 (1.2%) | 5 (3.1%) | 2 (3.4%) | 1 (7.7%) | 1 (33.3%) |
| **Physical activity level** |  |  |  |  |  |
| Low | 36 (13.9%) | 28 (17.5%) | 11 (18.6%) | 3 (23.1%) | 0 (0.0%) |
| Moderate | 133 (51.4%) | 75 (46.9%) | 37 (62.7%) | 7 (53.8%) | 2 (66.7%) |
| Vigorous | 78 (30.1%) | 43 (26.9%) | 8 (13.6%) | 1 (7.7%) | 0 (0.0%) |
| Unknown | 12 (4.6%) | 14 (8.8%) | 3 (5.1%) | 2 (15.4%) | 1 (33.3%) |
| **Academic factors** |  |  |  |  |  |
| **Year in medical school** |  |  |  |  |  |
| 4th year | 80 (30.9%) | 56 (35.0%) | 18 (30.5%) | 6 (46.2%) | 0 (0.0%) |
| 5th year | 101 (39.0%) | 48 (30.0%) | 13 (22.0%) | 4 (30.8%) | 0 (0.0%) |
| 6th year | 78 (30.1%) | 56 (35.0%) | 28 (47.5%) | 3 (23.1%) | 3 (100.0%) |
| **GPA of the previous year, median (IQR)** | 7.68 (7.38, 8.01) | 7.645 (7.3, 8) | 7.62 (7.33, 7.92) | 7.74 (7.47, 7.84) | 7.75 (7.4, 7.94) |
| **Failing a test in medical school** |  |  |  |  |  |
| Never | 127 (49.0%) | 68 (42.5%) | 23 (39.0%) | 5 (38.5%) | 1 (33.3%) |
| Ever | 132 (51.0%) | 92 (57.5%) | 36 (61.0%) | 8 (61.5%) | 2 (66.7%) |
| **Negative effects of night shifts on academic performance and quality of life** |  |  |  |  |  |
| No | 158 (61.0%) | 72 (45.0%) | 25 (42.4%) | 6 (46.2%) | 1 (33.3%) |
| Yes | 101 (39.0%) | 88 (55.0%) | 34 (57.6%) | 7 (53.8%) | 2 (66.7%) |
| **Academic motivation profile** |  |  |  |  |  |
| Self-determined | 225 (86.9%) | 128 (80.0%) | 38 (64.4%) | 5 (38.5%) | 1 (33.3%) |
| Non-self-determined | 34 (13.1%) | 32 (20.0%) | 21 (35.6%) | 8 (61.5%) | 2 (66.7%) |
| **Suicidal ideation** |  |  |  |  |  |
| No | 258 (99.6%) | 150 (93.8%) | 43 (72.9%) | 4 (30.8%) | 1 (33.3%) |
| Yes | 1 (0.4%) | 10 (6.3%) | 16 (27.1%) | 9 (69.2%) | 2 (66.7%) |

Grade point average (GPA) is presented as median (interquartile range); other variables are presented as number (percentage).
