# Supplementary material for: The prevalence of depression and associated risk factors among medical students: An untold story in Vietnam
Source: PLoS One. 2019 Aug 20;14(8):e0221432. doi: 10.1371/journal.pone.0221432 (PMC6701769; doi:10.1371/journal.pone.0221432)
Supplement: S1 File — (PDF) [file pone.0221432.s002.pdf]

VIMED Project – Clinical Learning Survey
Mã số nghiên cứu: [__][__][__][__]
Người thu thập:________________________
Ngày thu thập: _____ / _____ / _____

BỘ CÂU HỎI NGHIÊN CỨU

1. THÔNG TIN CÁ NHÂN

STT	Câu hỏi	Câu trả lời		Ghi chú
A01	Giới tính của bạn	Nam<br>Nữ	1<br>2	
A02	Bạn là sinh viên năm thứ mấy?	Năm 1<br>Năm 2<br>Năm 3<br>Năm 4<br>Năm 5<br>Năm 6	1<br>2<br>3<br>4<br>5<br>6	Khoanh tròn vào câu trả lời phù hợp
A03	Hiện tại bạn đang sống với gia đình (bố mẹ, anh chị em hoặc ông bà, cô chú…) hay ở trọ (thuê nhà, ký túc xá)?	Sống với gia đình<br>Ở trọ 1 mình<br>Ở trọ với bạn	1<br>2<br>3	
A04	Điểm trung bình trung học tập của bạn trong năm học vừa qua?	Học kỳ I: [__] . [__] [__]<br>Học kỳ II: [__] . [__] [__]		Bạn vui lòng tham khảo **tờ bảng điểm** điều tra viên phát để điền số điểm chính xác<br><br>Bạn có thể điền **“99”** vào các ô mà bạn không nhớ chính xác
A05	Điểm trung bình thi lâm sàng của bạn trong các năm học trước	Năm 3: [__] . [__] [__]<br>Năm 4: [__] . [__] [__]<br>Năm 5: [__] . [__] [__]		
A06	Trong các năm vừa qua bạn có phải thi lại **<u>lâm sàng</u>** lần nào không?	1. Không<br>2. Có (vui lòng ghi rõ số môn)<br>Năm thứ 3: [__]<br>Năm thứ 4: [__]<br>Năm thứ 5: [__] [__]		
A07	Trong các năm vừa qua bạn có phải thi lại **<u>lý thuyết</u>** lần nào không?	1. Không<br>2. Có (vui lòng ghi rõ số môn)<br>Năm thứ 1: [__] [__]<br>Năm thứ 2: [__] [__]<br>Năm thứ 3: [__] [__]<br>Năm thứ 4: [__] [__]<br>Năm thứ 5: [__] [__]		

2. TÌNH HÌNH HỌC LÂM SÀNG

2.1. Động lực học tập

Bạn hãy sử dụng thang điểm dưới đây để đánh giá sự phù hợp của các nhận định sau về lý do tại sao bạn học đại học y.

Hoàn toàn không phù hợp		Phù hợp 1 chút	Phù hợp mức trung bình		Rất phù hợp	Hoàn toàn phù hợp
1	2	3	4	5	6	7

STT	Câu hỏi	1	2	3	4	5	6	7
**Tại sao bạn học đại học y?**								
B01	Bởi vì nếu chỉ có tấm bằng tốt nghiệp phổ thông thì sau này tôi sẽ không tìm được một công việc có mức lương cao							
B02	Bởi vì tôi cảm thấy thích thú và hài lòng khi học được những điều mới							
B03	Bởi vì tôi nghĩ rằng giáo dục đại học sẽ giúp tôi chuẩn bị tốt hơn cho nghề nghiệp tôi chọn							
B04	Vì những cảm xúc mạnh mẽ tôi trải qua khi tôi nói về những ý tưởng của mình với người khác							
B05	Thành thực mà nói ,tôi không biết tại sao tôi học y, tôi thật sự cảm thấy mình đang lãng phí thời gian ở trường đại học							
B06	Vì tôi cảm thấy thích thú khi vượt qua chính bản thân mình trong học tập							
B07	Để chứng tỏ với bản thân mình rằng tôi có thể tốt nghiệp đại học							
B08	Để sau này có được một công việc danh giá hơn							
B09	Vì tôi cảm thấy thích thú khi khám phá ra những điều mới mẻ mà tôi chưa từng biết trước đây							
B10	Bởi vì cuối cùng học đại học sẽ giúp tôi tham gia vào thị trường việc làm trong lĩnh vực mà tôi thích							
B11	Vì tôi cảm thấy thích thú khi tôi đọc những bài viết của những tác giả thú vị							
B12	Tôi đã từng có những lý do chính đáng cho việc học đại học, nhưng bây giờ tôi đang băn khoăn liệu tôi có nên tiếp tục hay không							
B13	Vì tôi cảm thấy thích thú với cảm giác vượt qua chính bản thân mình							

Hoàn toàn không phù hợp			Phù hợp 1 chút		Phù hợp mức trung bình		Rất phù hợp		Hoàn toàn phù hợp
1	2	3	4	5	6	7			

STT	Câu hỏi	1	2	3	4	5	6	7
	khi đạt được một thành tựu cá nhân							
B14	Bởi vì thực tế là khi tôi thành công ở trường đại học, tôi cảm thấy mình quan trọng							
B15	Bởi vì tôi muốn có một cuộc sống tốt về sau							
B16	Vì tôi cảm thấy thích thú khi được mở mang kiến thức về các môn học mà tôi thấy hấp dẫn							
B17	Bởi vì học đại học cho tôi thêm một sự lựa chọn tốt hơn khi định hướng nghề nghiệp							
B18	Vì tôi cảm thấy thích thú khi tôi hoàn toàn say mê những gì một số tác giả viết ra							
B19	Tôi không thể trả lời tại sao tôi lại đi học đại học và nói một cách thẳng thắn, tôi không quan tâm đến vấn đề này							
B20	Vì tôi cảm thấy hài lòng trong quá trình tôi hoàn thành những công việc học thuật khó							
B21	Để thể hiện cho bản thân tôi thấy tôi là một người thông minh							
B22	Để sau này được trả lương cao hơn							
B23	Bởi vì học đại học cho phép tôi tiếp tục học nhiều thứ tôi thích							
B24	Bởi vì tôi tin rằng việc học tập trong một vài năm tới sẽ giúp cải thiện khả năng làm việc của tôi							
B25	Vì cảm giác “lâng lâng” mà tôi có khi đọc về các chủ đề thú vị khác nhau							
B26	Tôi không biết; tôi không thể hiểu tôi đang làm gì ở trường đại học							
B27	Bởi vì trường đại học cho tôi trải nghiệm cảm giác thoả mãn cá nhân khi theo đuổi sự xuất sắc trong học tập							
B28	Bởi vì tôi muốn thể hiện cho bản thân tôi thấy tôi có thể thành công trong việc học tập							

2.2. Tình hình trực lâm sàng

STT	Câu hỏi	Câu trả lời		Ghi chú
F01	Số buổi trực nhiều ảnh hưởng đến sức khỏe và khả năng học tập của bạn	Rất đồng ý<br>Đồng ý<br>Trung bình<br>Không đồng ý<br>Rất không đồng ý	4<br>3<br>2<br>1<br>0	

3. ĐÁNH GIÁ TÂM LÝ

3.1. Stress trong trường đại học

Bạn hãy đưa ra nhận định của mình về các mệnh đề sau và đánh dấu X vào ô phù hợp

STT	Câu hỏi	Rất không đồng ý	Không đồng ý	Trung bình	Đồng ý	Rất đồng ý
H01	Trường y khiến tôi tăng cảm giác mình không được ai biết đến và bị cô lập với các sinh viên khác					
H02	Tôi lo ngại mình sẽ không thể chịu đựng nhiều giờ học kéo dài và những trách nhiệm liên quan tới việc học và thực hành lâm sàng					
H03	Tôi không biết nhà trường mong đợi điều gì ở tôi					
H04	Việc học y chiếm phần lớn thời gian của tôi và tôi có quá ít thời gian cho các hoạt động khác					
H05	Tôi lo ngại mình sẽ không thể nắm vững toàn bộ lượng kiến thức y khoa					
H06	Trường y này đang thúc đẩy bác sĩ nâng cao chuyên môn nhưng khiến họ mất đi cá tính và thời gian cho các mối quan tâm khác					
H07	Trường y cạnh tranh hơn tôi đã nghĩ					
H08	Rất nhiều nhân viên của trường có quan điểm là sinh viên thực tập nên tự tìm cách xoay sở khi gặp khó khăn					
H09	Phần lớn sinh viên cảm thấy việc thành công ở trong trường y không phụ thuộc vào hệ thống quản lý và không phải do hệ thống đó					
H10	Trường y lạnh lùng, không tương thân tương ái và quan liêu không cần thiết					
H11	Các quyết định về các môn học tự chọn và tua lâm sàng được dựa trên thông tin lấy từ những sinh viên khóa trước, không phải từ nhà trường					
H12	Trường y mang tính đe dọa hơn là thách thức					
H13	Các khoản chi tiêu cá nhân là một mối lo ngại của tôi					

3.2. Đánh giá tâm lý khác

STT	Câu hỏi	Không có	Vài ngày	Quá nửa số ngày trong tuần	Hầu như mọi ngày
**Trong <u>2 TUẦN VỪA QUA</u>, bạn thường xuyên gặp phải bất cứ một trong các vấn đề sau ở mức độ nào? Bạn hãy đánh dấu X vào ô phù hợp<br>PHQ-9**					
I01	Giảm thích thú hoặc hài lòng trong công việc				
I02	Cảm thấy chán nản, trầm cảm hoặc tuyệt vọng				
I03	Khó ngủ hoặc khó ngủ lâu, hoặc ngủ quá nhiều				
I04	Cảm thấy mệt mỏi hoặc gần như kiệt sức				
I05	Chán ăn hoặc ăn quá nhiều				
I06	Cảm thấy bản thân mình tồi tệ — hoặc cảm thấy mình vô dụng hay đã làm bản thân hoặc gia đình thất vọng				
I07	Khó tập trung vào việc gì đó, chẳng hạn như đọc báo hoặc xem tivi				
I08	Di chuyển hoặc nói chậm đến mức người khác có thể nhận thấy? Hoặc ngược lại — quá bồn chồn hoặc bứt rứt đến nỗi chuyển động nhiều hơn bình thường				
I09	Nghĩ rằng mình nên chết quách đi cho xong hay muốn tự làm mình tổn thương theo cách nào đó				
**GAD-7**					
I10	Cảm thấy bồn chồn, lo lắng hoặc bất an				
I11	Không thể ngưng hoặc kiểm soát sự lo lắng				
I12	Lo lắng quá mức về nhiều thứ				
I13	Khó thư giãn				
I14	Bứt rứt đến mức khó ngồi yên				
I15	Trở nên dễ bực bội hoặc cáu kỉnh				
I16	Cảm thấy lo lắng như thể điều gì khủng khiếp có thể xảy ra				
**Nếu bạn đã đánh dấu <u>bất cứ</u> vấn đề nào, đâu là mức độ mà chúng gây <u>khó khăn</u> cho bạn khi: làm việc, trông nom nhà cửa hoặc hòa hợp với người khác?**					
I17	☐ Không khó chút nào	☐ Hơi khó	☐ Rất khó	☐ Cực kỳ khó	

4. ĐÁNH GIÁ VỀ LỐI SỐNG

4.1. Hoạt động thể lực

STT	Câu hỏi	Câu trả lời	Ghi chú
Chúng tôi quan tâm đến việc tìm ra những hình thức hoạt động thể lực mà mọi người làm trong cuộc sống hàng ngày. Các câu hỏi sẽ hỏi bạn về thời gian bạn đã sử dụng cho việc hoạt động thể lực trong vòng **7 NGÀY QUA**. Xin vui lòng trả lời từng câu hỏi mặc dù bạn có cho rằng bạn là một người ít hoạt động thể lực. Nghĩ tới các hoạt động thể lực tại nơi làm việc, công việc nhà, làm vườn đi lại, các hoạt động trong thời gian rảnh rỗi, giải trí và thể dục thể thao.			
Hãy nghĩ tới tất cả các hoạt động **nặng nhọc** mà bạn đã thực hiện trong vòng **7 ngày qua**. Các hoạt động **nặng nhọc** bao gồm các hoạt động đòi hỏi gắng sức về mặt thể lực và làm bạn **thở hổn hển** hơn. *Chỉ* tính các hoạt động thể lực kéo dài **ít nhất 10 phút** một lần.			
J01	Trong vòng **7 ngày qua**, có bao nhiêu ngày bạn đã thực hiện các hoạt động thể lực **nặng nhọc** như nâng vật nặng, đào đất, thể dục nhịp điệu hoặc đạp xe nhanh?	Không hoạt động thể lực nặng nhọc ☐<br>Số ngày trong tuần: [__]	→ **J03**
J02	Một trong những ngày đó, bạn thường dành bao nhiêu thời gian cho các hoạt động thể lực **nặng nhọc**?	Số giờ trong ngày: [__] [__]<br>Số phút trong ngày: [__] [__]<br>Không biết/Không chắc [__]	
Nghĩ tới các hoạt động thể lực **vừa phải** mà bạn đã thực hiện trong vòng **7 ngày qua**. Hoạt động thể lực **vừa phải** bao gồm các hoạt động đòi hỏi cố gắng thể lực vừa phải và làm bạn **thở nhanh** hơn bình thường. Chỉ tính các hoạt động thể lực kéo dài **ít nhất 10 phút** một lần			
J03	Trong vòng **7 ngày qua**, có bao nhiêu ngày bạn đã thực hiện các hoạt động thể **vừa phải** như nâng vật nhẹ, đạp xe ở tốc độ bình thường, chơi tennis đôi? Không tính đi bộ.	Không hoạt động thể lực vừa phải ☐<br>Số ngày trong tuần: [__]	→ **J05**
J04	Một trong những ngày đó, bạn thường dành bao nhiêu thời gian cho các hoạt động thể lực **vừa phải**?	Số giờ trong ngày: [__] [__]<br>Số phút trong ngày: [__] [__]<br>Không biết/Không chắc [__]	
Nghĩ tới thời gian mà bạn **đi bộ** trong **vòng 7 ngày qua**. Bao gồm ở nơi làm việc, ở nhà, khi **di chuyển** từ chỗ này đến chỗ kia hoặc bất cứ hoạt động đi bộ bạn thực hiện cho giải trí, thể dục thể thao hay các công việc trong lúc nhàn rỗi**.**			
J05	Trong vòng **7 ngày qua**, có bao nhiêu ngày bạn đã **đi bộ** kéo dài ít nhất 10 phút?	Không đi bộ ☐<br>Số ngày trong tuần: [__]	→ **J07**
J06	Một trong những ngày đó, bạn thường dành bao nhiêu thời gian **đi bộ**?	Số giờ trong ngày: [__] [__]<br>Số phút trong ngày: [__] [__]<br>Không biết/Không chắc [__]	
Câu cuối cùng hỏi về thời gian bạn sử dụng cho việc **ngồi** trong những ngày làm việc trong vòng **7 ngày qua**. Bao gồm thời gian dành cho công việc, ở nhà, trong lúc học tập và trong thời gian rảnh. Bao gồm cả thời gian dành cho việc ngồi bàn giấy, thăm bạn bè, đọc sách hay ngồi và nằm xem TV.			
J07	Trong vòng **7 ngày qua**, bạn dành bao nhiêu thời gian cho việc **ngồi** trong một **ngày làm việc**?	Số giờ trong ngày: [__] [__]<br>Số phút trong ngày: [__] [__]<br>Không biết/Không chắc [__]	

4.2. Đánh giá về hút thuốc

STT	Câu hỏi	Câu trả lời		Ghi chú
K01	**Hiện tại** bạn có đang hút thuốc lá hoặc thuốc lào không?	Không<br>Có	0<br>1	
K02	**Trước đây** bạn có bao giờ hút thuốc lá/thuốc lào thường xuyên không?<br>**(Ít nhất 02 điếu/ngày)**	Không<br>Có	0<br>1	→ **L01**

4.3. Đánh giá về sử dụng đồ uống có cồn

STT	Câu hỏi	Câu trả lời		Ghi chú
L01	Bạn có thường xuyên uống rượu hoặc bia nhiều hơn **1 lần/tháng** HOẶC **12 lần/năm** hay không?	Không<br>Có	0<br>1	→ **Kết thúc**

Xin chân thành cảm ơn bạn đã tham gia phỏng vấn!
